# Editorial: Cytokines and chemokines in lymphoma

**DOI:** 10.3389/fimmu.2025.1749323

**Published:** 2025-11-26

**Authors:** Marshall E. Kadin, Patrizia Fuschiotti, Alvise Sernicola

**Affiliations:** 1 Department of Pathology, University of Virginia School of Medicine, Charlottesville, VA, United States; 2 Department of Medicine, Division of Rheumatology and Clinical Immunology, University of Pittsburgh School of Medicine, Pittsburg, PA, United States; 3 Department of Medicine, University of Padua, Padua, Italy

**Keywords:** lymphoma, cytokines, chemokines, T-cell lymphoma, B-cell lymphoma, lymphomageneis, lymphoproliferative disorders

Cytokines, chemokines and their receptors play a major role in the pathogenesis and clinical manifestations of human lymphomas. IL-13 is a growth factor for tumor cells in mycoides fungoides (1) and Hodgkin lymphoma (2) and is produced by tumor cells of breast implant associated anaplastic large cell lymphoma (3). TGF-b is a profibrotic cytokine that promotes collagen fibrosis in nodular sclerosing Hodgkin lymphoma (4). TGF- b is a negative regulator of CD30^+^ anaplastic lymphoma and other T-cell lymphomas (5, 6). IL-6 promotes growth and is a therapeutic target in diffuse large B cell lymphoma and plasma cell myeloma (7, 8). IL-5 produced by Reed-Sternberg cells is associated with eosinophilia of Hodgkin lymphoma (9). CCR4, the receptor for TARC is expressed on tumor cells and is a target for mogamulizumab in treatment of refractory or relapsed cutaneous T-cell lymphomas (10). CD1a^+^ histiocytes (Langerhans cells) and CD3^+^ T cells produced large amounts of cytokines in histiocytosis X; IL-2, IL-4, IL-5, and TNF-a are produced exclusively by T cells, whereas only IL-1a is produced by Langerhans cells (5). The IL-1R pathway is a potential target in anaplastic large cell lymphomas (11).

In the context of lymphomagenesis, a type 2 immune profile is typical of mycosis fungoides (MF) and Sezary syndrome (SS), the most frequent subtypes of cutaneous T cell lymphoma (CTCL) (12). Key cytokines of type 2 immunity – including IL-4, IL-13, IL-31, and the thymic stromal lymphopoietin – have been implicated to different degrees in the pathogenesis of these cutaneous entities as well as in their associated pruritus (13, 14). In-depth understanding on the contribution of these cytokines to the onset and progression of CTCL may provide a rationale for the investigation of targeted therapeutic agents in this specific setting (1). Moreover, cutaneous B cell lymphomas constitute an overall rare subgroup and a current area of unmet therapeutic need (15). Lymphoid tissue-associated chemokines, as well as vascular addressins, are crucial regulators in both physiologic homing of B cells and in B cell lymphomagenesis in the skin. These signals support the development of indolent subtypes of primary cutaneous marginal zone lymphoma and primary cutaneous follicle center lymphoma by interacting with surface chemokine receptors, such as CXCR4 or CXCR5 receptors, that are broadly expressed across B cells in these disorders (16, 17). Studies comparing indolent and aggressive subtypes of cutaneous B cell lymphoma as well as these cutaneous entities and their nodal counterparts are needed to identify molecular differences that may account for the peculiar and mostly favorable behavior associated to cutaneous lymphomas. Advancing our pathogenetic understanding in this setting may provide potential targets for drug discovery in the setting of lymphoproliferative disorders in the skin (2, 18, 19).

IL-10 is an immunosuppressive cytokine. IL-6 is a pro-inflammatory cytokine which promotes growth of B lymphocytes. Wen et al. found high IL-10 concentrations in central nervous system (CNS) fluid and serum of patients with diffuse large B-cell lymphoma (DLBCL) with secondary CNS involvement. They reported that an increased IL-10/IL-6 ratio in pretreatment serum of patients with DLBCL was an independent predictor of secondary CNS involvement and progression free survival. Their results underscore the potential of integrating inflammatory cytokine biomarkers with established clinical indices to refine risk stratification. IL-10 has been reported to be increased in vitreous humor of patients with intra-ocular lymphoma, anatomically linked to the CNS, consistent with their finding. Interestingly, Valenzuela et al. reported IL-10 and IL-6/IL-10 as predictive biomarkers for treatment response in non-infectious uveitis (20). In a clinically unrelated disease, we reported the IL-10/IL-6 ratio is increased in peri-implant effusions of women who develop anaplastic large cell T-cell lymphoma around textured breast implants (21).

CTCLs are easily accessible for study of mechanisms of interaction of tumor cells with reactive immune cells. Gugliemo et al. reviewed the contribution of chemokine receptors CCR4, CCR7, CCR8, CCR10, CXCR3, and CXCR4 in MF/SS to disease evolution of skin-based MF and leukemic SS. They explored the role of chemokines and their receptors in the hypothesized transition of CTCL from a Th1 to a Th2 immune profile in tumor progression with emphasis on their therapeutic potential. Some applications of chemokine receptors to clinical practice are listed below. Mogamulizumab, a humanized anti-CCR4 monoclonal antibody, is primarily used as a second-line agent in advanced-stage MF; loss of CCR4 renders patients refractory to mogamulizumab therapy (22). CCR7, which mediates lymph node homing, is expressed at higher levels in tumor progression of MF but has not yet been targeted therapeutically (23). CCR8 is upregulated on malignant CD4^+^ T cells in SS and tumor-stage MF, contributing to their epidermotropism and persistence in skin (24). Because CCR8 is not typically expressed in benign lymphoproliferative disorders, it can be useful in the diagnosis of CTCL (25). Readers are encouraged to read the paper of Gugliemo et al. in full to gain more complete insight into the chemokines in CTCL.

The management of patients with SS is a current challenge, even in the era of targeted therapeutics such as mogamulizumab, due to expected relapses and development of resistance. Subjects with SS therefore are frequently administered sequential and combinatory regimens to improve therapeutic outcome (26). Extracorporeal photopheresis (ECP) is a beneficial and safe strategy consisting in photosensitization of the peripheral blood with psoralen and subsequent UVA irradiation which is performed extracorporeally (27). The results of a study by Melchers et al. showed that a single ECP session opposes the Th2 imbalance and enhances anti-tumor immunity, supporting an early addition of ECP to combination regimens for the treatment of SS. In their study involving six SS patients, the authors highlighted the immunologic changes that occur in the plasmatic cytokine profile and peripheral blood mononuclear cell (PBMC) composition after one session of ECP (3). Following treatment, IL-15 and IL-17, together with IL1R and TLR3, were increased with a pro-inflammatory profile. Conversely, IL13R1 and IL13R2 were decreased, down-regulating the Th2 polarization typical of SS. Modifications affecting chemokine signaling included increased CCL7 and CXCL12 and decreased CXCR5. Moreover, factors inducing apoptosis, Fas and TRAIL, were increased (28). Finally, malignant CD4^+^ CD26^-^ cells were decreased or stable post-ECP.

The OX40-OX40L axis plays a key role in CTCL tumorigenesis, mainly by promoting proliferation and survival of malignant T cells through MAPK/ERK signaling (29, 30). The OX40-OX40L pathway is also critical for controlling cytokine production by T cells, especially in upregulating Th2 cytokines (31), notably IL-5 and IL-13, which are important pro-oncogenic factors in CTCL (1, 29, 32). In this Research Topic, Papadavid et al. used a chick embryo spontaneous metastasis model (33) and CTCL cell lines in which OX40 expression was knocked-out by CRISPR-Cas9 to demonstrate that the OX40-OX40L axis facilitates proliferation, tumor implantation and transendothelial migration of malignant T lymphocytes. They also show that the OX40-OX40L axis promotes distant organ metastasis through the immune regulatory actions of M2 macrophages and lymphangiogenesis induced by VEGF-C. The authors conclude that OX40 represents a key driver of CTCL progression, promoting tumor growth and metastasis through ERK activation and they validate the chick embryo model as a potential preclinical tool for therapeutic testing.
